# Exosomal miR-543 Inhibits the Proliferation of Ovarian Cancer by Targeting IGF2

**DOI:** 10.1155/2022/2003739

**Published:** 2022-03-29

**Authors:** Shupei Zhang, Diling Pan, Shaoyu Zhang, Qiumei Wu, Lan Zhen, Shihuang Liu, Jingjing Chen, Rong Lin, Qiuhua Hong, Xiangqin Zheng, Huan Yi

**Affiliations:** ^1^Gynaecology Oncology, Fujian Maternity and Child Health Hospital College of Clinical Medicine for Obstetrics & Gynecology and Pediatrics, Fujian Medical University, Fujian, China; ^2^Fujian Key Laboratory of Women and Children's Critical Diseases Research, Fujian Maternity and Child Health Hospital, Fuzhou 350001, China; ^3^Obstetrics and Gynaecology, Fuding General Hospital, Fuding, China

## Abstract

**Objective:**

Ovarian cancer (OvCa) is the most lethal gynaecological malignancy worldwide. We aimed to illustrate the potential function and molecular mechanism of exosomal microRNA-543 (miR-543) in the oncogenesis and development of OvCa.

**Methods:**

Differentially expressed microRNAs in exosomes derived from OvCa cell lines were identified by bioinformatic analysis and verified by RT-PCR. Cell proliferation ability was estimated by clonogenic and 5-ethynyl-2′-deoxyuridine assays in vitro and in vivo. Potential involved pathways and targets of exosomal miRNAs were analysed using DIANA and verified by pyrosequencing, glucose quantification, dual-luciferase reporter experiments, and functional rescue assays.

**Results:**

Bioinformatic analysis identified miR-543 and its potential target genes involved in the cancer-associated proteoglycan pathway. The expression of miR-543 was significantly decreased in exosomes derived from OvCa cell lines, patient serum, and OvCa tissues, while the mRNA levels of insulin-like growth factor 2 (IGF2) were increased. Furthermore, the overexpression of miR-543 resulted in the suppression of OvCa cell proliferation in vitro and in vivo. Moreover, miR-543 was significantly negatively correlated with IGF2 in OvCa tissues in comparison with paracarcinoma tissues. Notably, upregulation of miR-543 led to increased cell supernatant glucose levels and suppressed cell growth, which was rescued by overexpression of IGF2.

**Conclusions:**

Exosomal miR-543 participates in the proteoglycan pathway to suppress cell proliferation by targeting IGF2 in OvCa.

## 1. Introduction

Ovarian cancer (OvCa) is the most lethal gynaecological malignancy globally, with an unimproved 5-year survival rate of less than 45% and a 10-year survival rate of less than 30% during the last 30 years [1–3]. Maintenance therapy, which has been developed from targeted treatment and is a newly implemented but important approach following debulking surgery and chemotherapy, is an essential and promising component of the whole-course management of OvCa, especially at the late stage (FIGO IIB-IV) [4]. Currently, antiangiogenic drugs [5, 6] and poly (ADP-ribose) polymerase inhibitors (PAPRi) [7] are the two main strategies of maintenance therapies. Eligible OvCa patients undergoing the appropriate maintenance therapy have a significantly improved prognosis [7, 8]. However, the heterogeneity of OvCa and resistance to maintenance therapies acquired in advanced disease pose major obstacles to the universal use of this therapeutic strategy. Therefore, a better understanding of OvCa pathophysiology and the exploration of new potential diagnostic and therapeutic targets for overcoming the current issues are required.

Herein, increasing evidence reveals the significance of exosomes in OvCa pathogenesis and progression. Epithelial ovarian cancer (EOC) is the most common type of OvCa (accounting for approximately 80% of cases) and has the highest mortality among all types of OvCa [9, 10]. Exosomes are endosome-packaged, 30-150 nm lipid bilayer extracellular vesicles that are produced by most cells, including cancer cells and immune cells [11]. In addition, exosomes function as important regulatory signaling transporters between parental invasive cancer cells and target cells and are involved in cellular energy metabolism [12], angiogenesis [13, 14], protumorigenic signaling pathways [15], immune escape [16], proliferation, and metastasis [17]. Specifically, exosomes are enriched in extracellular RNA (miRNAs and mRNAs) and proteins and express exosomal-specific markers (CD9, CD63, and TSG101) but lack glycolytic enzymes, extracellular DNA, and cytoskeletal components [18]. Therefore, exosomes likely regulate tumour energy metabolism by delivering extracellular RNAs from tumour cells to target cells.

However, the mechanisms linking OvCa metabolic dysregulation and exosomal miRNAs are incompletely understood. This manuscript focuses on revealing correlations between exosomal miRNAs and energy metabolism in OvCa and investigates possible molecules as tumour diagnostic and therapeutic targets.

## 2. Materials and Methods

### 2.1. miRNA Microarray Data

To study miRNAs in OvCa-derived exosomes, we used the keyword “ovarian cancer exosome miRNA” to search the Gene Expression Omnibus (GEO) database [19] and found one miRNA microarray dataset, GSE76449 [20]. One normal human ovarian surface epithelial cell line (HIO180), six different invasive OvCa cell lines, namely, HEYA8_MDR (multidrug-resistant), A2780_CP20 (cisplatin-resistant), and SKOV3_TR (Taxol-resistant), and the chemosensitive OvCa cell lines HEYA8, A2780_PAR, and SKOV3_ipl and their exosomal samples were analysed by 4.0 miRNA Affymetrix chips. Two biological repeats were employed in each sample. The series matrix and platform files were downloaded as TXT files.

### 2.2. Identification of Differentially Expressed miRNAs (DE-miRNAs)

Data were investigated by using GEO2R (http://www.ncbi.nlm.nlh.gov/geo/geo2r/). Significant miRNAs with the thresholds of ∣log fold change (logFC) | >0.58 and *P* value < 0.05 were subjected to cluster analysis. To further determine the reliability of the bioinformatic analysis, the overlapping miRNAs were shown using a Venn diagram. The DE-miRNAs were further selected by their differential expression in both cancer cell-isolated exosomes vs. normal cell-isolated exosomes and OvCa cells vs. normal cells. Heatmaps and volcano plots of DE-miRNAs were generated using R software.

### 2.3. Prediction of Key Targeted Genes by DE-miRNAs

The online analysis tool DIANA (http://diana.imis.athena-innovation.gr/DianaTools/) predicted DE-miRNA-targeted genes and Kyoto Encyclopedia of Genes and Genomes (KEGG) pathways. DIANA-MicroT-CDS was used to predict target genes of DE-miRNAs, and mirPath v.3 was used for the DE-miRNA pathway analysis. To further investigate the targeted genes in the glucose-related metabolic pathway, we annotated, visualized, and integrated them by using the STRING database (http://string-db.org) to construct a protein-protein interaction (PPI) network. The DAVID online database was applied to analyse key genes in terms of KEGG pathways and Gene Ontology (GO) terms, which included the biological process (BP), molecular function (MF), and cellular component (CC) ontologies.

### 2.4. Association of Targeted Genes, Patient Prognosis, and Cancer Stages

GEPIA2 (http://gepia2.cancer-pku.cn/#index) is an online survival analysis tool that was used to identify genes associated with the age, histological grade, stage, treatment, and overall survival (OS) of OvCa. Kaplan-Meier survival curves were constructed for the high- and low-expression groups.

### 2.5. Cell Culture and Human Tissues

Human OvCa cell lines (SKOV3, COCI, CAOV3, OVCAR3, SW626, OV90, and HEY) and a human normal ovarian cell line (IOSE80) were obtained from the Shanghai Cancer Institute. OV90 cells were cultured in MCDB105/medium 199 complete medium (ScienCell, Shanghai, China). SKOV3 cells were cultured in McCoy's 5A medium (HyClone, Logan, USA), and the other seven OvCa cell lines were cultured in DMEM (HyClone, Logan, USA). All the media were supplemented with 10% (volume/volume) foetal bovine serum (FBS) (Gibco, Invitrogen, USA) and 1% (volume/volume) penicillin/streptomycin (P/S). All cell lines were incubated in a 37°C humidified incubator with 5% CO_2_. We enrolled 30 patients pathologically diagnosed with EOC and 30 normal controls at Fujian Provincial Maternal and Children Hospital from September 2016 to September 2020. Samples of cancer-adjacent tissues and OvCa lesions were collected for methylation analysis. All experiments involving human tissue samples in this study were approved by the Ethics Committee of Fujian Provincial Maternal and Children Health Hospital, and written informed consent was obtained.

### 2.6. Cell Transfection

A lentiviral vector carrying miR-543 (3′-GTCCGGACTCAGATCTCGAGCTTGACGGTTGCCCGGTGCGCATCAG GACCCATGTGCTCTCAG-5′) was transfected into SKOV3 and HEY cells in the logarithmic growth phase following the manufacturer's protocol (Invitrogen, California, USA). The expression of miR-543 was assessed by using quantitative reverse transcription PCR (qRT-PCR) to verify the transfection efficiency.

### 2.7. Exosome Purification and Identification

Serum samples were obtained from all participants after fasting for 8 hours. Exosomes were extracted from patient serum using a total exosome isolation kit following the manufacturer's protocol (Invitrogen, California, USA). Additionally, the culture medium of transfected cells was collected and centrifuged to remove cell debris and other impurities. Then, the exosomes were extracted and purified according to the instructions. Finally, a Zetaview instrument was utilized for transmission electron microscopy (TEM) and nanoparticle tracking analysis (NTA) to verify the exosomes. For TEM, exosomes were fixed successively with 4% glutaraldehyde and 1% osmium tetroxide (OsO4). The specimens were dehydrated in graded ethanol solutions from 50% to 100% for 10 minutes and then embedded in EPON resin. The embedded tissue was cut into 100 nm thin sections and restained with uranyl acetate and lead citrate. TEM was utilized for exosome observation and imaging.

### 2.8. qRT-PCR

Total RNA was extracted by lysis of target cells and exosomes with the TRIzol reagent (Invitrogen, California, USA) according to the manufacturer's protocol. The purity and concentration of total isolated RNA were determined by using a UV-Vis spectrophotometer Q5000 (Quawell, California, USA) at a wavelength of 260 nm. Total isolated RNA was then subjected to RT with the microRNA RT kit (Promega, Wisconsin, USA) using a two-step process according to the instructions from the manufacturer. qRT-PCR was performed with GoTaq Green Master Mix (Promega, Wisconsin, USA). U6 (Sangon Biotech, China) was used as an internal reference to standardize miRNA concentrations (forward primer sequence: 5′-CTCGCTTCGGCAGCACA-3′, reverse primer sequence: 5′-AACGCTTCACGAATTTGCGT-3′). The forward primer sequence of miR-543 was 5′-CGAAACATTCGCGGTGCA-3′, and the reverse primer sequence was 5′-AGTGCAGGGTCCGAGGTATT-3′. The miRNA expression value was determined using an ABI7500 instrument purchased from Applied Biosystems. The 2^−ΔΔCt^ method was used to calculate the expression of target miRNAs and genes to generate relevant standard curves.

### 2.9. Cell Counting Kit-8 (CCK8) Assay

The cell proliferation assay was performed with the CCK-8 (Beyotime, Shanghai, China) assay following the manufacturer's instructions.

### 2.10. Colony Formation Assay

The transfected cells were resuspended, diluted, and inoculated into a 6-well plate at a density of 1 × 10^3^/ml in serum-free medium. After 14 days, the colonies were fixed with 4% paraformaldehyde, stained with 0.5% crystal violet, imaged, and counted.

### 2.11. EdU Proliferation Assay

To measure ovarian cancer cell proliferation, a 5-ethynyl-2′-deoxyuridine (EdU) assay was performed following the manufacturer's protocol (US EVERBRIGHT, Suzhou, China). SKOV3 and HEY cells transfected with NC or miR-543 were plated in 6-well plates at a density of 1 × 10^6^ cells/well and then treated with 10 nM docetaxel for 48 hours. Harvested cells were washed twice with PBS and incubated in 10 *μ*mol/L EdU (US EVERBRIGHT, Suzhou, China) diluted with serum-free DMEM for 2 hours. Then, the cells were fixed, subjected to DNA staining, and imaged using fluorescence microscopy, and five random fields were calculated.

### 2.12. Analysis of Glucose Concentration in Cell Supernatants

The glucose concentration in the cell supernatant was determined by utilizing a Glucose Assay Kit (Rongsheng Bio, Shanghai, China) according to the manufacturer's instructions.

### 2.13. WB Analysis

The target cells were lysed with the appropriate volume of RIPA buffer (Beyotime, Nanjing, China) supplemented with PMSF (Thermo Fisher, Waltham, USA). Then, the concentration of the extracted protein was quantified with a BCA Protein Assay Kit (Beyotime, Nanjing, China) by plotting a standard curve. After SDS-PAGE separation, the protein samples were transferred to PVDF membranes (Millipore, Bedford, MA). The membranes were successively blocked with 5% BSA, incubated overnight with primary antibodies against IGF1, IGF2, and GAPDH (Cell Signaling Technology, Massachusetts, USA) at 4°C, and incubated for 1-2 hours with HRP-conjugated goat anti-rabbit or anti-mouse secondary antibody (ImmunoWay, Newark, USA). The bands were visualized by using a DAB HRP Color Development Kit (Beyotime, Nanjing, China) and imaged by the Fluor Chem R chemiluminescence system (ProteinSimple, California, USA). The intensity of the bands was measured and analysed quantitatively with GAPDH as the control.

### 2.14. Subcutaneously Implanted Tumour Model

BALB/c female nude mice aged approximately 5 weeks were purchased from Charles River Laboratories (Zhejiang, China) and housed in pathogen-free cages. Then, miR-543-up and negative control HEY cells resuspended in 0.1 ml PBS were injected subcutaneously into the right flanks of nude mice at a density of 1 × 10^6^/ml suspended in 200 *μ*l PBS. The tumour volume was estimated and recorded once a week by the formula volume = *π*/6 × length × width × height. The tumours were excised from the mice and imaged after 8 weeks. The procedure was approved by the Ethics Committee for Animal Experiments of Fujian Province Maternal and Children Hospital.

### 2.15. Dual-Luciferase Reporter Assay

The amplified 3′-UTR of IGF2 was cloned upstream of the firefly luciferase gene (Promega, Wisconsin, USA) to construct the wild-type luciferase reporter plasmid. Meanwhile, the mutant plasmid of the IGF2 3′-UTR was constructed by mutating the predicted miR-543 binding site using a mutagenesis kit (Gene, Shanghai, China). The luciferase activity of cells transfected for 48 hours was tested with the Dual-Glo Luciferase Reporter Assay System (Promega, Wisconsin, USA). All steps followed the corresponding manufacturer's instructions.

### 2.16. Pyrosequencing

Pyrosequencing was performed to detect the methylation distribution of OvCa tissues compared with adjacent normal tissues. First, PyroMark Assay Design 2.0 (Qiagen, Frankfurt, Germany) was used to design primers for sequencing and amplification. Then, genomic DNA was subjected to bisulfite conversion to convert unmethylated cytosine into thymine, and the target region was amplified with specific primers by using a PyroMark® PCR kit (Qiagen, Frankfurt, Germany) according to the manufacturer's instructions. Finally, pyrosequencing of single-stranded DNA was performed in PyroMark Q96/48 ID (Qiagen, Frankfurt, Germany) following the protocol provided by the manufacturer, and the corresponding extent of methylation was determined by analysing the percentage of cytosine/thymine in the target segment.

### 2.17. Immunohistochemical (IHC) Assay

Tissues from subcutaneously implanted tumours in the mouse model were sliced into 4 *μ*M sections and prepared for IHC staining. Antibody dilutions were 1 : 50 for the Ki67 mouse polyclonal antibody (ImmunoWay, Texas, USA). The IHC procedure was performed following the manufacturer's recommendations.

### 2.18. Statistical Analysis

SPSS software version 21.0 (SPSS Inc., NY, USA) was used for statistical analysis. Student's *t*-test was performed to compare the differences between two independent-sample groups. *P* < 0.05 was considered to indicate a statistically significant difference.

## 3. Results

### 3.1. Key Targeted Genes in the Proteoglycans in the Cancer Pathway

**Figure 1:**
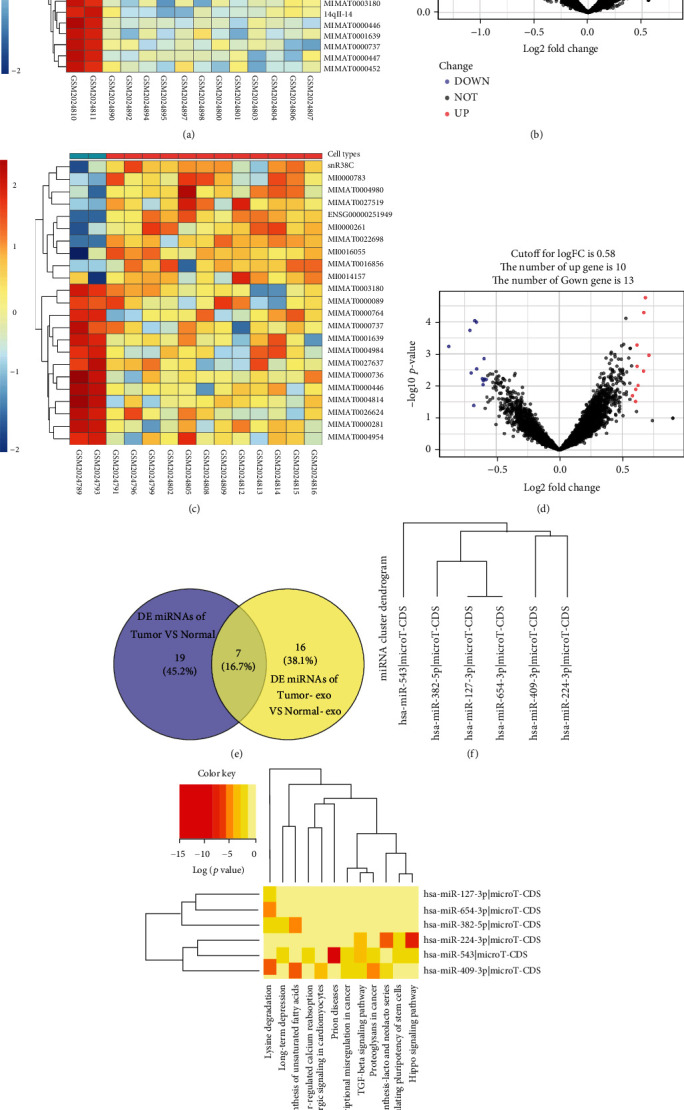
Profiles of significant DE-miRNAs in OvCa cells and OvCa-derived exosomes. Heatmaps and volcano plots indicate the levels of differential miRNA expression in OvCa cell lines (a, b) and OvCa-derived exosomes (c, d). (e) Analysis of DE-miRNAs shown in the Venn diagram. (f) Cluster dendrogram of possible DE-miRNAs involved in metabolism-related pathways predicted by DIANA tools. (G) Heatmaps of DE-miRNAs involved in metabolism-related pathways using DIANA tools. Abbreviation: OvCa: ovarian cancer; DE: differentially expressed.

GSE71449 was downloaded and processed from the GEO database. Significantly DE-miRNAs were identified and are shown in a heatmap and volcano plots, respectively (Figures 1(a)–1(d)). A Venn diagram indicated that of the 24 DE-miRNAs, 7 were significantly downregulated in OvCa cells vs. normal cells and their derived exosomes (Figure 1(e)).

Metabolism is vital in the progression of OvCa [21]. To identify metabolism-related pathways, we used DIANA tools and found that the DE-miRNAs mainly regulated 12 pathways (miRNA-4876-3p was excluded due to a lack of annotation in the database) (Figure 1(f)). The DE-miRNA-enriched metabolism-related pathways included biosynthesis of unsaturated fatty acids, proteoglycans in cancer, glycosphingolipid biosynthesis-lacto and neolacto series, and other pathways (Figure 1(g)). The results demonstrated that miR-543 was involved in the highest number of pathways and proteoglycans in OvCa and was selected for further study for its crucial role in tumour proliferation and angiogenesis [22]. Overall, a total of 26 genes were included because they were the predicted miR-543 targeted genes that regulated proteoglycans in OvCa.

**Figure 2:**
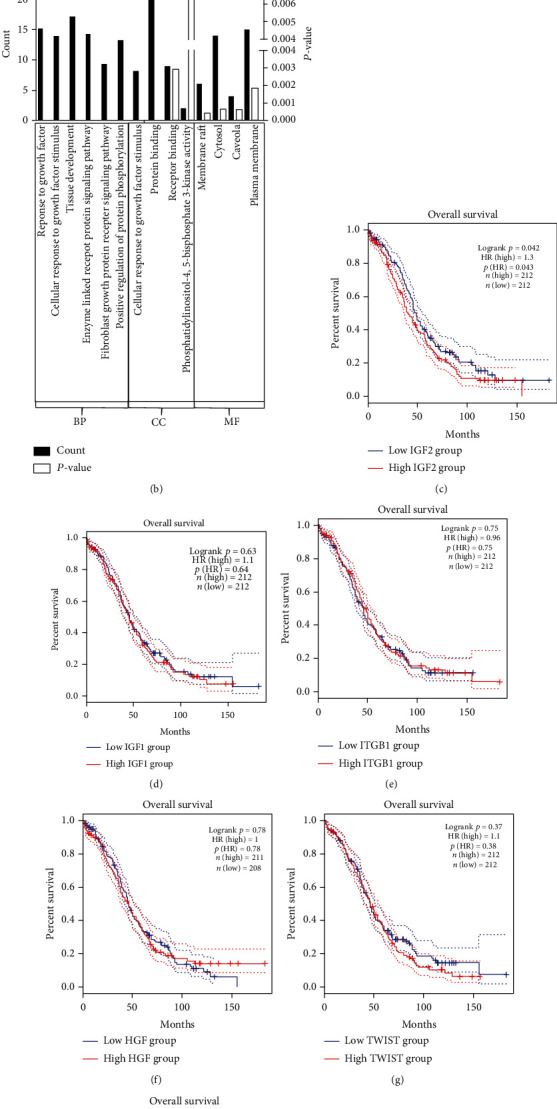
Predicted key targeted genes of miR-543 involved in the proteoglycan pathway by using bioinformatic analysis. (a) Protein-protein interaction (PPI) network of predicted targets of miR-543. (b) Gene Ontology (GO) analysis of predicted key targeted genes of miR-543. (c–i) Correlation between predicted target genes of miR-543 and patient survival of OvCa. Abbreviations: CC: cellular component; MF: molecular function; BP: biological process.

To elucidate the unknown genes unique to EOC involved in glucose metabolism, we constructed an interaction network of the 26 predicted miR-543 target genes by applying the STRING online database. Consequently, the interlinked network between genes from the predicted genes closely related to the proteoglycans in the cancer pathway is illustrated in Figure 2(a). Furthermore, GO functional enrichment was performed for these genes. All results were ranked by statistically enriched score [−log (*P* value)], and the top hits of each category are displayed in Figure 2(b). In terms of biological processes, the top 3 enriched terms were response to growth factor, cellular response to growth factor stimulus, and tissue development. In addition, cellular response to fibroblast growth factor stimulus, protein binding, and receptor binding were the top 3 enriched terms in the cellular component analysis, while membrane raft, cytosol, and caveola were the top enriched terms in the molecular function analysis. Apart from proteoglycans in cancer, pathways in cancer and focal adhesion were ranked in the top three pathways in the KEGG analysis (Table 1).

### 3.2. Correlation between Key Genes, Patient Clinicopathological Factors, and Survival

To determine the correlation between the patient prognosis and stage in patients with EOC, Kaplan-Meier survival curves and stage plots comparing the expression of the 24 predicted target genes of miR-543 and patient prognosis and stage in TCGA cohort were generated. IGF2 (*P* = 0.042) was identified as a strong indicator of the clinical survival time of EOC patients (Figure 2(c)). Besides, ITGB1, HGF, TWIST1, IGF-1, PPP1R12A, and BRAF exhibited no significant correlations with overall survival time (Figures 2(d)–2(i)). However, none of the genes manifested statistically significant differences in the tumour stage, patient age, or tumour grade.

### 3.3. Overexpression of miR-543 Inhibits the Proliferation of EOC

**Figure 3:**
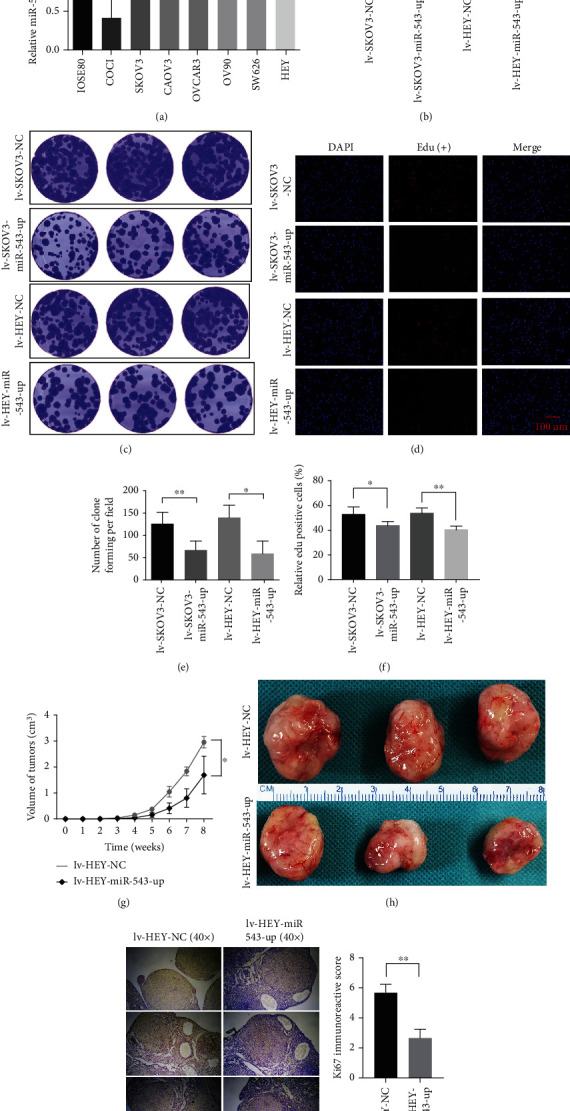
In vitro and in vivo functional assays of miR-543 in OvCa. (a) Relative miR-543 quantitative expression in normal ovarian cell lines and wild-type OvCa cell lines. (b) The stable upregulated expression of miR-543 was tested in SKOV3 and HEY cells by RT-PCR. Then, the stable clone forming (c, e) and Edu (d, f) assays revealed a significantly decreased proliferation ability after stable overexpression of miR-543. (g, h) In vivo assays showed that the tumour volume of the OvCa subcutaneous xenograft of lv-HEY-miR-543-up cells was significantly lower than that of the control cells. (i, j) Ki67 staining was significantly decreased in the subcutaneous xenograft of lv-HEY-miR-543-up cells compared with that in controls. Abbreviation: lv: lentivirus; Edu: 5-ethynyl-2′-deoxyuridine. ^∗^*P* < 0.05 vs. control (unpaired *t*-test), ^∗∗^*P* < 0.01 vs. control (unpaired *t*-test), and ^∗∗∗^*P* < 0.001 vs. control (unpaired *t*-test).

Tumour invasion and colony formation are vital and final malignant behaviours during EOC progression. To test whether miR-543 is required for cell invasion and proliferation, we examined the expression of miR-543 in wild-type normal ovarian cells and OvCa cells (Figure 3(a)). Based on the results, we overexpressed miR-543 in SKOV3 and HEY cells because they had relatively low expression of miR-543 among the tested OvCa cells. Further experiments confirmed obvious overexpression of miR-543 in these two cell lines by stable transfection (Figure 3(b)). Furthermore, overexpression of miR-543 significantly decreased the proliferation rates of SKOV3 and HEY cells in colony formation assays (Figures 3(c) and 3(e)) and the suppressed proliferative function was explored in the Edu assay (Figures 3(d) and 3(f)).

We further investigated whether miR-543 also plays a proliferative suppressor role in EOC in vivo. Similar to the in vitro results, we observed that nude female mice injected with HEY cells overexpressing miR-543 presented obviously smaller tumours than those injected with control cells (Figures 3(g)–3(h)). The in vivo assays provided additional evidence that miR-543 plays a tumour suppressive role during EOC progression. Ki67, which represents the proliferation ability in vivo, was expressed at significantly lower levels by IHC after overexpressing miR-543 in a subcutaneous xenograft mouse model in comparison with that of controls (Figures 3(i) and 3(j)).

### 3.4. Exosomal miR-543 Is a Strong Indicator of OvCa

**Figure 4:**
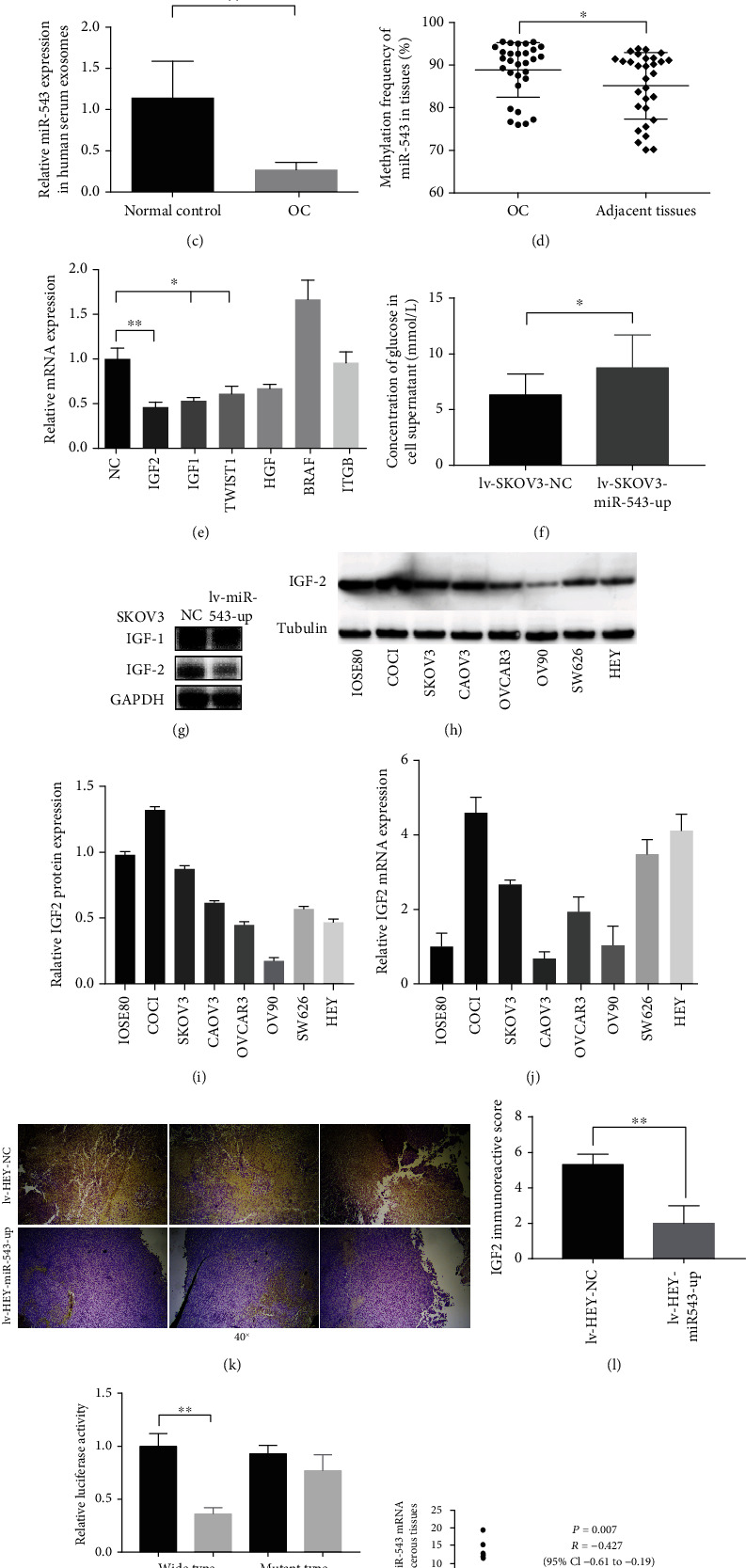
Regulatory network of miR-543 in inhibiting proliferation in EOC. (a) Similar to the bioinformatic analysis results, the expression of miR-543 was significantly lower in OvCa tissues than in normal ovarian tissues. (b) Exosomes derived from OvCa patient serum were verified by TEM and NTA. (c) The expression of miR-543 was also significantly lower in OvCa patient-derived exosomes than in normal control exosomes. (d) Compared to that in adjacent tissues, the methylation frequency of miR-543 in OvCa tissues was obviously higher. (e) The mRNA levels of predicted target genes of miR-543 that influenced survival were quantified by qRT-PCR in lv-SKOV-miR-543-up cells. (f) The glucose concentration in the cell supernatant was obviously increased in lv-SKOV-miR-543-up cells compared to control cells. (g) Although IGF1 and IGF2 are homologous proteins, WB assays showed that the protein level of IGF2 was significantly lower in lv-SKOV-miR-543-up cells than in control cells, while the IGF1 level remained the same between the two groups. (h–j) The protein and mRNA level of IGF2 in OvCa cell lines. (k, l) IGF2 was significantly decreased in upregulating miR-543 in the subcutaneous xenograft models than in controls. (m) The putative binding sites of miR-543 and IGF2 were predicted by using StarBase version 2.0 and verified by dual-luciferase reporter assays. (n) The mRNA levels of miR-543 and IGF2 in ovarian cancer tissues versus paracancerous tissues show a significantly negative correlation between them. Abbreviation: lv: lentivirus; OC: ovarian cancer. ^∗^*P* < 0.05 vs. control (unpaired *t*-test), ^∗∗^*P* < 0.01 vs. control (unpaired *t*-test).

Dataset (GSE71449) analysis showed that the level of miR-543 expression was significantly lower in exosomes derived from OvCa cells than in exosomes derived from normal ovarian cells. To confirm the differential expression in clinical samples, we next investigated the expression of miR-543 in exosomes derived from patients with EOC, EOC tissues, and the corresponding controls. The expression of miR-543 was significantly lower in EOC tissues (*n* = 60) than in normal ovarian tissues (*n* = 60) (*P* = 0.026, Figure 4(a)). Exosomes extracted from EOC patient serum and controls were tested by TEM and NTA.  Figure 4(b) shows that the exosomes were confirmed to be typical round-plate structures with sizes of 30-150 nm. Consistent with the findings in tissues, the exosomal level of miR-543 was significantly lower in EOC patients than in the normal ovary controls (*P* = 0.0047, Figure 4(c)).

### 3.5. Regulatory Network of miR-543 in Inhibiting Proliferation in EOC

Methylation of miRNAs is closely associated with tumour proliferation and is known to be increased in gastrointestinal cancer [23]. Therefore, we assessed the methylation frequency of miR-543 in EOC tissues and paired normal tissues. As shown in Figure 4(d), the methylation frequency of miR-543 was considerably higher in EOC tissues (96% ± 3%) than in adjacent tissues (93% ± 3%). Therefore, methylation was shown to downregulate miR-543 in EOC progression.

Bioinformatic analysis allowed us to identify potential targets of miR-543 that are associated with the proteoglycans in cancer pathway. After stable overexpression of miR-543 in SKOV3 cells, the mRNA levels of IGF2 (*P* = 0.0076), IGF1 (*P* = 0.022), and TWIST1 (*P* = 0.019) were notably decreased in comparison to those in the control cells, as demonstrated using RT-PCR assays (Figure 4(e)). IGF2 is an essential glucose regulatory factor that promotes the proliferation of several cancers [24, 25]. Because according to RT-PCR, the reduction degree of IGF2 was the most significant, we next tested the concentration of glucose in the supernatant of miR-543-overexpressing cells. As expected, the level of glucose was significantly higher in miR-543-overexpressing cells than in control cells (Figure 4(f)). We further performed the WB assay to demonstrate that miR-543 reduced IGF2 in SKOV3 cells at the protein level (Figure 4(g)). Figures 4(h)–4(l) show that IGF2 was relatively conversely expressed in OvCa cell lines and transplanted tumor in the mouse model at the protein and mRNA levels, respectively, compared with the expression of miR-543.

### 3.6. IGF2 Is Responsible for the miR-543-Mediated Suppression of Proliferation in EOC

Only one predicted binding site of miR-543 in the 3′-UTR of IGF2 mRNA (5736-5742 nt) was found. Subsequently, to confirm that IGF2 is a direct target of miR-543, we conducted a luciferase reporter assay and found a 56.3% reduction in luciferase activity when SKOV3 cells were cotransfected with miR-543 compared with the control cells, suggesting that miR-543 directly targets IGF2 (Figure 4(m)). Moreover, we quantified IGF2 and miR-543 mRNA levels in ovarian cancer and paracancerous tissues. These results showed that miR-543 was significantly negatively correlated with IGF2 (Figure 4(n)).

**Figure 5:**
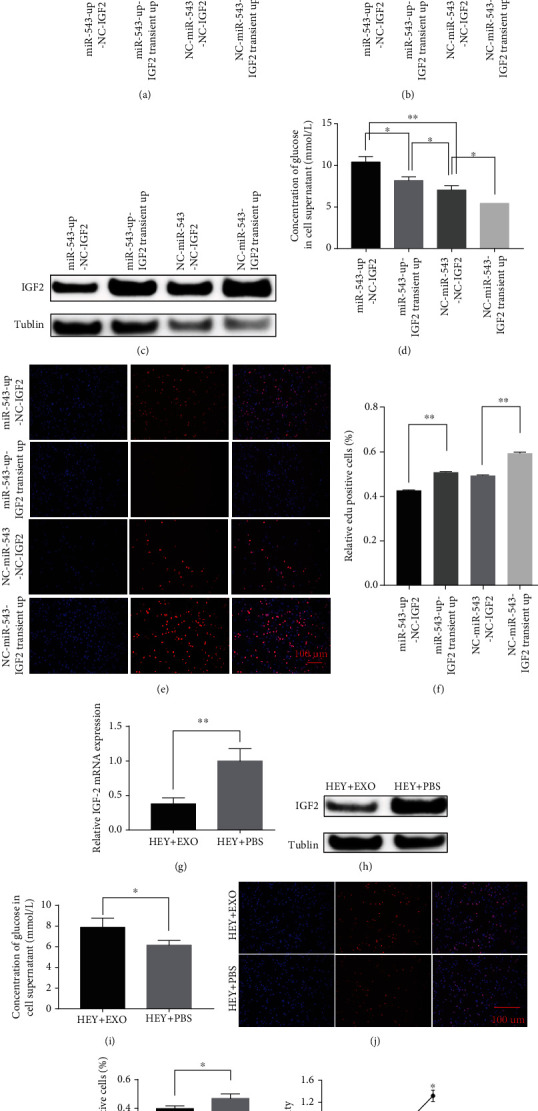
Rescue assays of IGF2 in lv-HEY-miR-543-up cells. (a) The transiently upregulated expression of IGF2 in lv-HEY-miR-543-up cells and controls was verified by qRT-PCR. (b, c) The expression of IGF2 among lv-HEY-miR-543-up-NC-IGF2, lv-HEY-miR-543-up-IGF2 transient up, lv-NC-miR-543-NC-IGF2, and lv-NC-miR-543-IGF2 transient up at mRNA and protein levels. (d) The concentration of glucose in the cell supernatant was significantly decreased after rescue of the expression of IGF2. In addition, the Edu (e, f) proliferative abilities were all rescued by upregulating the expression of IGF2 in lv-HEY-miR-543-up cells. IGF2 was significantly downregulated after being cocultivated with OvCa-derived exosomes than that with controls. Meanwhile, the glucose secretion was significantly increased, and the proliferation ability in OvCa cells was significantly decreased after the treatment of exosome-derived miR-543 in tumour cells (g–l). Abbreviation: lv: lentivirus; Edu: 5-ethynyl-2′-deoxyuridine. ^∗^*P* < 0.05 vs. control (unpaired *t*-test), ^∗∗^*P* < 0.01 vs. control (unpaired *t*-test).

To prove that downregulation of IGF2 is essential for miR-543-mediated suppression of proliferation in EOC, we next performed functional rescue assays. HEY cells overexpressing miR-543 (miR-543-up cells) were cotransfected with an IGF2-overexpressing plasmid (Figure 5(a)). The difference in IGF2 expression at the protein level was analysed in Figures 5(b) and 5(c). Conversely, the concentration of glucose in the cellular supernatant was significantly lower in the cotransfected cells than in the control cells (Figure 5(d)). The glucose supply is very important for tumour proliferation. Consequently, upregulation of IGF2 in miR-543-up HEY cells reversed the suppressive effect of miR-543 in the EdU assay (Figures 5(e)–5(f)) in vitro.

Besides, 50 *μ*g/ml of exosomes extracted from CAOV3 cells (expressing the most miR-543 in Figure 3(a)) was added into HEY cell lines and cocultured for 24 hours. At mRNA and protein levels, Figures 5(h)–5(i) show that the expression of IGF2 was significantly downregulated after being cocultivated with OvCa-derived exosomes compared with controls. Meanwhile, the glucose secretion was significantly increased and the proliferation ability in OvCa cells was significantly decreased after the treatment with exosome-derived miR-543 in tumour cells (Figures 5(g)–5(l)).

**Figure 6:**
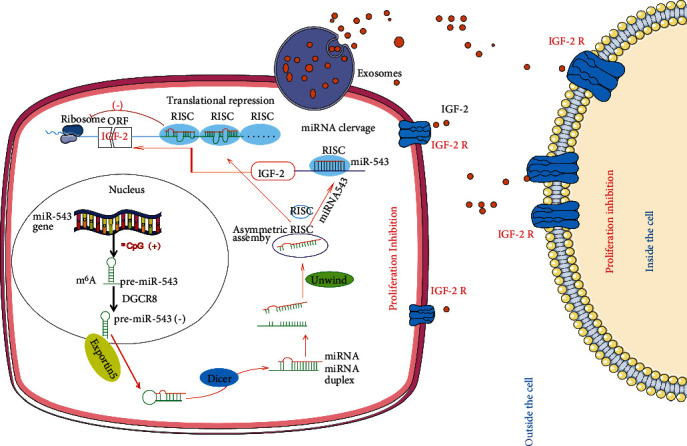
Summary of the underlying mechanism by which miR-543 is involved in inhibiting OvCa proliferation. miR-543 was significantly decreased in OvCa tissues by methylation and was thus reduced in OvCa-derived exosomes. During exosome-mediated communication between tumour cells, distant target cells, and the tumour microenvironment, IGF2, as the direct target of miR-543, was significantly decreased, leading to significantly suppressed proliferation of OvCa. Abbreviation: RISC: RNA-induced silencing complex; DGCR8: DiGeorge syndrome critical region gene 8.

These functional rescue results indicated that IGF2 is a bona fide target of miR-543 in the suppression of OvCa proliferation, and the associated mechanism is shown in Figure 6.

## 4. Discussion

OvCa is a highly heterogeneous cancer with a poor 5-year survival rate of less than 45% [3]. To improve the unsatisfactory clinical outcome of OvCa, there is a pressing need to identify more effective drug targets and cancer-associated molecular mechanisms. To date, studies have revealed that exosomal miRNAs show a range of cancer-regulating properties, including the control of cancer growth. Exosomal miRNAs that are differentially expressed in cancer result in abnormal proteoglycan pathways, thus leading to tumour growth and metastasis [26]. In the current study, database analysis revealed that the expression of miR-543 was significantly lower in exosomes derived from OvCa cells than in those derived from normal ovarian cells. Furthermore, predicted miR-543 targets were enriched in the proteoglycans in the cancer pathway. Both in vitro and in vivo functional assays indicated that the miR-543 mimic significantly suppressed the invasive and proliferative abilities of OvCa cells. We also observed that methylation reduced miR-543 in OvCa tissues compared to adjacent normal tissues. Importantly, IGF2, which is involved in the proteoglycan pathway, was identified as a direct target of miR-543 and rescued miR-543-related suppression of proliferation in OvCa cells. These findings provide additional evidence of a suppressive role of miR-543 and indicate the diagnostic and therapeutic value of miR-543 for OvCa progression.

The basic and terminal hallmark of tumour development is proliferation, in which reprogramming of the energy metabolism pathway occurs [27]. Reprogramming of energy metabolism is a complex process that includes metabolism-related enzymes and membrane transporters. Exosomes carry molecular cargo to transfer signals from tumour cells into the tumour and tumour microenvironment, thus regulating metabolism and consequently proliferation. Recent experimental assays have shown that exosomes released by tumour cells into the tumour microenvironment are an important source of functional RNAs and proteins but lack “free circulating” DNA, glycolytic enzymes, and cytoskeletal components [18]. Therefore, “free circulating” RNAs in tumour-derived exosomes likely regulate glycolytic enzymes and cytoskeletal constituents by targeting metabolic and cytoskeletal genes. Lactate derived from glucose or glycogen breakdown is an important energy supplement for tumour proliferation [28]. Most published studies have investigated exosomal miRNAs derived from cultured tumour cells, which may not be consistent with those derived from OvCa patients. In the current study, we initially provided evidence that miR-543 secreted by OvCa patient exosomes was downregulated and promoted proliferation by regulating the target IGF2 to participate in proteoglycan pathways, which regulate metabolism and cytoskeletal synthesis [29].

Similar to our results, miR-543 has been identified as a tumour suppressor in pancreatic cancer, colorectal cancer [30], breast cancer [31], glioma [32], and cervical cancer [33]. However, other studies have indicated an oncogenic role of miR-543 in digestive and urinary system cancers [34]. These controversial findings indicate that miR-543 is involved in a number of pathways in different cancer diseases. Yu et al. reported that miR-543 was downregulated at the cellular and tissue levels in OvCa [35, 36]. Moreover, mechanistic analysis showed that lncRNA PVT1 and placental growth factor significantly reduced miR-543 expression, and SERPINI1 and TWIST1 were the target genes. Currently, there is no experimental evidence that shows the role of methylation and target genes of miR-543 involved in metabolism in OvCa. In the current study, we demonstrated that methylation downregulated miR-543 in OvCa tissues and exosomes, and IGF2 is a critical direct downstream metabolic target involved in tumour proliferation [19, 37].

Epigenetic alterations, such as changes in miRNA-mediated processes and RNA methylation, are involved in proliferation in various types of invasive cancers [38]. This is the first study to show the high level of miR-543 methylation in OvCa, and its delivery by exosomes leads to IGF2 dysfunction. IGF2 binding activates IGF1R and IGF2R and is associated with aberrant glucose metabolism and proteoglycan dysregulation, which is responsible for cancer development [39]. Proteoglycans are important molecules that participate in cytoskeletal processes, such as the synthesis of the extracellular matrix and cell membrane. Furthermore, high expression of IGF2 was correlated with poor clinical outcome, chemoresistance, and increased proliferation and migration of OvCa [40–42]. Drugs that block IGF2 and decrease glucose levels, such as metformin, have become a promising approach to prevent and treat cancer [43, 44]. Similar results were observed in the current study, whereby miR-543 overexpression in OvCa cells significantly increased the concentration of glucose in the medium and suppressed proliferation, while rescuing IGF2 expression in miR-543 mimic-transfected OvCa cells resulted in decreased glucose in the medium and increased cell growth.

In conclusion, our findings provide evidence for OvCa-secreted exosomes that downregulate miR-543 by methylation and thus rescue the inhibitory effect on IGF2 to promote proliferation. These findings improve our understanding of the involvement of miR-543 in metabolism and cytoskeletal biology and identify miR-543 as a candidate for clarifying OvCa development and a crucial therapeutic and diagnostic biomarker.

## Figures and Tables

**Table 1:** KEGG pathways.

Pathway ID	Pathway description	Count in gene set	FDR (false discovery rate)
05205	Proteoglycans in cancer	23	8.86*e* − 43
05200	Pathways in cancer	14	9.67*e* − 18
04510	Focal adhesion	12	1.44*e* − 16
05218	Melanoma	8	5.57*e* − 13
04015	Rap 1 signaling pathway	10	7.77*e* − 13
04810	Regulation of actin cytoskeleton	10	7.88*e* − 13
04012	ErbB signaling pathway	8	1.49*e* − 12
05215	Prostate cancer	8	1.58*e* − 12
05211	Renal cell carcinoma	7	2.21*e* − 11
05100	Bacterial invasion of epithelial cancer	7	5.19*e* − 11

## Data Availability

The datasets used and/or analyzed during the current study are available from the corresponding authors on reasonable request.

## References

[1] Jacobs I. J., Menon U., Ryan A. (2016). Ovarian cancer screening and mortality in the UK Collaborative Trial of Ovarian Cancer Screening (UKCTOCS): a randomised controlled trial. *Lancet*.

[2] Labidi-Galy S. I., Papp E., Hallberg D. (2017). High grade serous ovarian carcinomas originate in the fallopian tube. *Nature Communications*.

[3] Dinca A. L., Birla R. D., Dinca V. G., Marica C., Panaitescu E., Constantinoiu S. (2020). Prognostic factors in advanced ovarian cancer - a clinical trial. *Chirurgia*.

[4] Konecny G. E., Kristeleit R. S. (2016). PARP inhibitors for *BRCA1/2*-mutated and sporadic ovarian cancer: current practice and future directions. *British Journal of Cancer*.

[5] Khosravi-Shahi P., Cabezon-Gutierrez L. (2012). Antiangiogenic drugs in the treatment of advanced epithelial ovarian cancer. *Anti-Cancer Agents in Medicinal Chemistry*.

[6] Yi S., Zeng L., Kuang Y. (2017). Antiangiogenic drugs used with chemotherapy for patients with recurrent ovarian cancer: a meta-analysis. *Oncotargets and Therapy*.

[7] Madariaga A., Bonilla L., McMullen M., Oza A. M., Lheureux S. (2020). Efficacy and safety updates of poly ADP-ribose polymerase (PARP) inhibitor maintenance in ovarian cancer from ASCO 2020. *International Journal of Gynecological Cancer*.

[8] Armstrong D. K., Alvarez R. D., Bakkum-Gamez J. N. (2019). NCCN guidelines insights: ovarian cancer, version 1.2019. *Journal of the National Comprehensive Cancer Network*.

[9] Jia X., Liu X., Li M. (2018). Potential tumor suppressing role of microRNA-545 in epithelial ovarian cancer. *Oncology Letters*.

[10] Lu Q., Qu H., Lou T., Liu C., Zhang Z. (2020). CK19 promotes ovarian cancer development by impacting on Wnt/*β*-catenin pathway. *Oncotargets and Therapy*.

[11] Theodoraki M. N., Yerneni S. S., Hoffmann T. K., Gooding W. E., Whiteside T. L. (2018). Clinical significance of PD-L1^+^ exosomes in plasma of head and neck cancer patients. *Clinical Cancer Research*.

[12] Klinke D. J., Kulkarni Y. M., Wu Y., Byrne-Hoffman C. (2014). Inferring alterations in cell-to-cell communication in HER2+ breast cancer using secretome profiling of three cell models. *Biotechnology and Bioengineering*.

[13] Yi H., Ye J., Yang X. M., Zhang L. W., Zhang Z. G., Chen Y. P. (2015). High-grade ovarian cancer secreting effective exosomes in tumor angiogenesis. *International Journal of Clinical and Experimental Pathology*.

[14] Zhang X., Sheng Y., Li B., Wang Q., Liu X., Han J. (2021). Ovarian cancer derived PKR1 positive exosomes promote angiogenesis by promoting migration and tube formation in vitro. *Cell Biochemistry and Function*.

[15] Bussard K. M., Mutkus L., Stumpf K., Gomez-Manzano C., Marini F. C. (2016). Tumor-associated stromal cells as key contributors to the tumor microenvironment. *Breast Cancer Research*.

[16] Yuan Y., Wang L., Ge D. (2021). Exosomal O-GlcNAc transferase from esophageal carcinoma stem cell promotes cancer immunosuppression through up-regulation of PD-1 in CD8^+^ T cells. *Cancer Letters*.

[17] Takasugi M., Okada R., Takahashi A., Virya Chen D., Watanabe S., Hara E. (2017). Small extracellular vesicles secreted from senescent cells promote cancer cell proliferation through EphA2. *Nature Communications*.

[18] Jeppesen D. K., Fenix A. M., Franklin J. L. (2019). Reassessment of exosome composition. *Cell*.

[19] Brouwer-Visser J., Huang G. S. (2015). IGF2 signaling and regulation in cancer. *Cytokine & Growth Factor Reviews*.

[20] Feng Y., Hang W., Sang Z. (2019). Identification of exosomal and non‑exosomal microRNAs associated with the drug resistance of ovarian cancer. *Molecular Medicine Reports*.

[21] Zhao S., Zhang X., Shi Y. (2020). MIEF2 over-expression promotes tumor growth and metastasis through reprogramming of glucose metabolism in ovarian cancer. *Journal of Experimental & Clinical Cancer Research*.

[22] Iozzo R. V., Schaefer L. (2015). Proteoglycan form and function: a comprehensive nomenclature of proteoglycans. *Matrix Biology*.

[23] Konno M., Koseki J., Asai A. (2019). Distinct methylation levels of mature microRNAs in gastrointestinal cancers. *Nature Communications*.

[24] Liu L., Li X. (2020). A review of IGF1 signaling and IGF1-related long noncoding RNAs in chemoresistance of cancer. *Current Cancer Drug Targets*.

[25] Wang X., Zhu Q., Lin Y. (2017). Crosstalk between TEMs and endothelial cells modulates angiogenesis and metastasis via IGF1-IGF1R signalling in epithelial ovarian cancer. *British Journal of Cancer*.

[26] Ibrahim S. A., Hassan H., Gotte M. (2014). MicroRNA regulation of proteoglycan function in cancer. *The FEBS Journal*.

[27] Hanahan D., Weinberg R. A. (2011). Hallmarks of cancer: the next generation. *Cell*.

[28] Ippolito L., Morandi A., Giannoni E., Chiarugi P. (2019). Lactate: a metabolic driver in the tumour landscape. *Trends in Biochemical Sciences*.

[29] Zhang H. B., Zeng Y., Li T. L., Wang G. (2020). Correlation between polymorphisms in IGF2/H19 gene locus and epithelial ovarian cancer risk in Chinese population. *Genomics*.

[30] Su D. W., Li X., Chen J., Dou J., Fang G. E., Luo C. J. (2020). MiR-543 inhibits proliferation and metastasis of human colorectal cancer cells by targeting PLAS3. *European Review for Medical and Pharmacological Sciences*.

[31] Wang H., Huang Y., Yang Y. (2020). LncRNA PVT1 regulates TRPS1 expression in breast cancer by sponging miR-543. *Cancer Management and Research*.

[32] Zhang Y., An J., Pei Y. (2020). LncRNA SNHG6 promotes LMO3 expression by sponging miR-543 in glioma. *Molecular and Cellular Biochemistry*.

[33] Liu X., Gan L., Zhang J. (2019). miR-543 inhibites cervical cancer growth and metastasis by targeting TRPM7. *Chemico-Biological Interactions*.

[34] Zhou C., Zhao X., Duan S. (2021). The role of miR-543 in human cancerous and noncancerous diseases. *Journal of Cellular Physiology*.

[35] Qu C., Dai C., Guo Y., Qin R., Liu J. (2020). Long non-coding RNA PVT1-mediated miR-543/SERPINI1 axis plays a key role in the regulatory mechanism of ovarian cancer. *Bioscience Reports*.

[36] Yu Q., Zhang Z., He B., Wang H., Shi P., Li Y. (2020). MiR-543 functions as tumor suppressor in ovarian cancer by targeting TWIST1. *Journal of Biological Regulators and Homeostatic Agents*.

[37] Murphy S. K., Huang Z., Wen Y. (2006). Frequent IGF2/H19 domain epigenetic alterations and elevated IGF2 expression in epithelial ovarian cancer. *Molecular Cancer Research*.

[38] Han T. S., Ban H. S., Hur K., Cho H. S. (2018). The epigenetic regulation of HCC metastasis. *International Journal of Molecular Sciences*.

[39] Hamamura K., Zhang P., Yokota H. (2008). IGF2-driven PI3 kinase and TGFbeta signaling pathways in chondrogenesis. *Cell Biology International*.

[40] Huang G. S., Brouwer-Visser J., Ramirez M. J. (2010). Insulin-like growth factor 2 expression modulates Taxol resistance and is a candidate biomarker for reduced disease-free survival in ovarian cancer. *Clinical Cancer Research*.

[41] Brouwer-Visser J., Lee J., McCullagh K., Cossio M. J., Wang Y., Huang G. S. (2014). Insulin-like growth factor 2 silencing restores taxol sensitivity in drug resistant ovarian cancer. *PLoS One*.

[42] Dong Y., Li J., Han F. (2015). High IGF2 expression is associated with poor clinical outcome in human ovarian cancer. *Oncology Reports*.

[43] Vella V., Nicolosi M. L., Giuliano M., Morrione A., Malaguarnera R., Belfiore A. (2019). Insulin receptor isoform A modulates metabolic reprogramming of breast cancer cells in response to IGF2 and insulin stimulation. *Cells*.

[44] Bosch-Barrera J., Hernandez A., Abella L. E. (2009). Insulin and insulin-like growth factor pathway, a new targeted therapy in oncology. *Anales del Sistema Sanitario de Navarra*.

